# Unifying Obstacle Detection, Recognition, and Fusion Based on the Polarization Color Stereo Camera and LiDAR for the ADAS

**DOI:** 10.3390/s22072453

**Published:** 2022-03-23

**Authors:** Ningbo Long, Han Yan, Liqiang Wang, Haifeng Li, Qing Yang

**Affiliations:** 1 Research Center for Humanoid Sensing, Zhejiang Lab, Hangzhou 311100, China; longningbo@zju.edu.cn (N.L.); wangliqiang@zju.edu.cn (L.W.); lihaifeng@zju.edu.cn (H.L.); 2 Science and Technology on Space Intelligent Control Laboratory, Beijing Institute of Control Engineering, Beijing 100094, China; yhustc@sina.com; 3 College of Optical Science and Engineering, Zhejiang University, Hangzhou 310027, China

**Keywords:** polarization-color-depth, stereo camera, non-repetitive scanning LiDAR, sensor fusion

## Abstract

The perception module plays an important role in vehicles equipped with advanced driver-assistance systems (ADAS). This paper presents a multi-sensor data fusion system based on the polarization color stereo camera and the forward-looking light detection and ranging (LiDAR), which achieves the multiple target detection, recognition, and data fusion. The You Only Look Once v4 (YOLOv4) network is utilized to achieve object detection and recognition on the color images. The depth images are obtained from the rectified left and right images based on the principle of the epipolar constraints, then the obstacles are detected from the depth images using the MeanShift algorithm. The pixel-level polarization images are extracted from the raw polarization-grey images, then the water hazards are detected successfully. The PointPillars network is employed to detect the objects from the point cloud. The calibration and synchronization between the sensors are accomplished. The experiment results show that the data fusion enriches the detection results, provides high-dimensional perceptual information and extends the effective detection range. Meanwhile, the detection results are stable under diverse range and illumination conditions.

## 1. Introduction

Advanced driver-assistance systems (ADAS) and upcoming autonomous cars have a strong need for an accurate perception [1] of the environment. In order to obtain robust and high-dimensional perceptual information, we propose a sensor fusion system which unifies the multiple target detection, recognition, and fusion based on the polarization color stereo camera and forward-looking light detection and ranging (LiDAR) sensor.

Over the past few years, computer vision (CV) has undergone a striking improvement, especially due to the development of deep learning [2,3,4,5]. The vision systems are able to provide appearance information. However, the RGB images lack necessary dimensional information, which makes it difficult for vision systems to fully perceive complex surrounding scenes. The stereo-vision systems could provide 3D information via a cost-effective solution. Nevertheless, the ranging results of remote objects derived from the stereo cameras are not accurate, and the objects without abundant textures are not robustly detected [6].

Apart from color and depth, polarization [7] and its imaging extend dimensional information for target detection. The polarization state of the light reflected from the object surface contains specific physical properties, which have been utilized to achieve the surface measurement. Compared with the previous polarization information acquisition method, by rotating polarization filters [8] or adding different polarization direction filters before the lens [9,10], the polarization camera, which has a pixel-level wire-grid polarizer structure on the CMOS is gradually employed [7].

However, the vision systems are sensitive to the variable environments. In contrast, the LiDAR detection results remain stabilized in varying illuminance. Simultaneously, the effective detection distance is much greater than the stereo camera, and the measurements maintain high accuracy [11]. Moreover, in recent years, the LiDAR technology has developed speedily, which makes it widely used in various fields. The forward-looking LiDAR [12,13] has the potential to deliver lifesaving and cost-saving benefits. Nevertheless, the LiDAR provides limited angular resolution.

As mentioned above, there are various advantages of fusing the polarization color stereo camera and forward-looking LiDAR [14,15]. The fusion mutually complements the drawbacks of each sensor, which improves the robustness of object detection and recognition in various situations [16,17]. The polarization color stereo camera provides the color images, depth images and the pixel-level polarization images. The color images are employed to achieve the object recognition, and the depth images provide the depth information of the detected objects. The pixel-level polarization images are endowed with the ability of water hazards detection. Meanwhile, the LiDAR provides the accurate distance information on every detected object in the scene. The sensor fusion system provides the complementary information and increases the perception module robustness under different circumstances.

**Figure 1:**
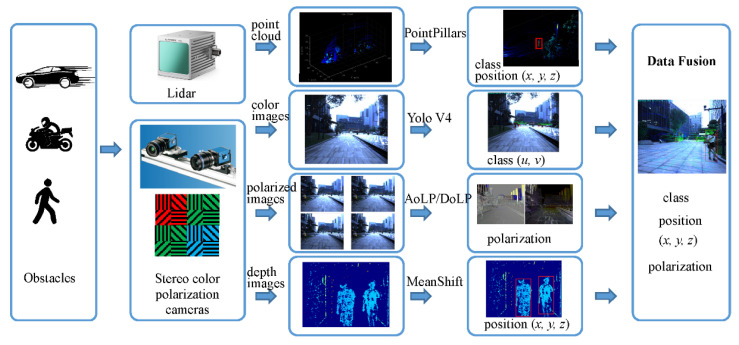
The proposed multiple target detection, recognition and fusion framework for ADAS.

In this paper, a unified targets detection, recognition and data fusion framework is proposed. The hardware platform comprises a stereo polarization color camera and a forward-looking LiDAR, as shown in Figure 1. Obstacles in the scene are detected by the stereo camera and LiDAR simultaneously. The pixel-level aligned polarization-color-depth data information is obtained by the stereo polarization color camera. The objects in the color images are recognized using deep learning, and the You Only Look Once v4 (YOLOv4) network [18] is adopted. The MeanShift [19] algorithm is applied onto the depth images to obtain the depth and position information of the obstacles. The pixel-level polarization information is used for the detection of slippery road surfaces and puddles in the urban environment. The category information of objects in the LiDAR point cloud is obtained using the PointPillars [20] network, then the more accurate range information compared to the depth image is taken. Thereafter, the detailed information about the current scene is obtained with the help of multiple sensor data fusion, which includes the objects’ class, position and polarization.

The remainder of this paper is structured as follows. In Section 2, a brief review of related work is provided. In Section 3, the proposed hardware and algorithm are subsequently explained in detail. Then, the experimental results are performed and discussed in Section 4. Finally, the relevant conclusions are drawn, and future works are expected in Section 5.

## 2. Related Work

In this section, the state-of-the-art multiple sensor data fusion is reviewed. LiDAR and camera are essential sensors in the perception module of the current self-driving car systems. The LiDAR system is good at extracting accurate depth information of objects. In comparison, the camera produces rich and detailed information. Many perception problems could be solved by fusing these two complementary sensors.

Gong et al. [21] proposed a frustum-based probabilistic framework for 3D object detection using the LiDAR and camera data fusion in a simultaneous localization and mapping (SLAM) environment, which solved the LiDAR point cloud sparse and noise problem. They verified the method using both backpack LiDAR dataset and the KITTI vision Benchmark Suite, and the results were outstanding.

Zhang et al. [22] developed a multiple sensor fusion experimental robot, which included a GNSS antenna and receiver, a stereo camera, a monocular camera, a Livox LiDAR and so on. The proposed system was tested on real scenarios, realized the localization and navigation function, and completed the dam crack identification and pedestrian detection.

Wang et al. [23] proposed a novel method termed High Dimensional Frustum PointNet for 3D object detection in the context of autonomous driving, which used the raw data from the camera, LiDAR and radar. Ku et al. [24] presented an aggregate view object detection network for autonomous driving. The LiDAR point clouds and RGB images were employed to generate features shared by the region proposal network (RPN) and the second stage detector network. The proposed RPN was able to generate reliable 3D object proposals with the help of multimodal feature fusion on high resolution feature maps. Caltagirone et al. [25] developed a novel multimodal system for road detection by fusing LiDAR and camera data, and the cross function convolutional neural network (FCN) architecture was introduced. Zhuang et al. [26] investigated a collaborative fusion scheme called perception-aware multi-sensor fusion to exploit perceptual information from two modalities that included appearance information from RGB images and the spatio-depth information from point clouds, which achieved the 3D semantic segmentation. An offline LiDAR and camera fusion method was proposed by Zhen et al. [27] to build dense and accurate 3D models. A road detection method, which uses the LiDAR and camera data fusion, was achieved by the Gu et al. [28] to obtain the range and color information simultaneously.

Plenty of related studies have been conducted to research the method of multiple sensor data fusion, which achieves object detection, SLAM, semantic segmentation and other functions. However, these studies neglect the application of the polarization information. Like wavelength, polarization is an essential property of light. The performance of existing machine vision could be improved when the polarization information is fully utilized. Compared with previous studies, our system provides the following advantages:

We propose a novel system that comprises a stereo polarization color camera and a forward-looking LiDAR, which achieves the target detection, recognition and multiple sensor data fusion;

In addition to the color and depth information, the pixel-level aligned polarization-color-depth data information with real time synchronous is also obtained, on the basis of the calibration and synchronization between the sensors;

The overall perception system becomes more robust to the varying conditions by the method of multiple sensor data fusion.

## 3. Methodology

In this section, we first present the system hardware configuration. Then, the object detection and recognition using the color images, the feature extraction of the polarization images, the object detection based on the depth images, and the object detection and recognition of the point cloud are introduced. After that, the calibration between the LiDAR and the stereo camera is accomplished. Finally, the data fusion is presented.

### 3.1. Sensors

**Figure 2:**
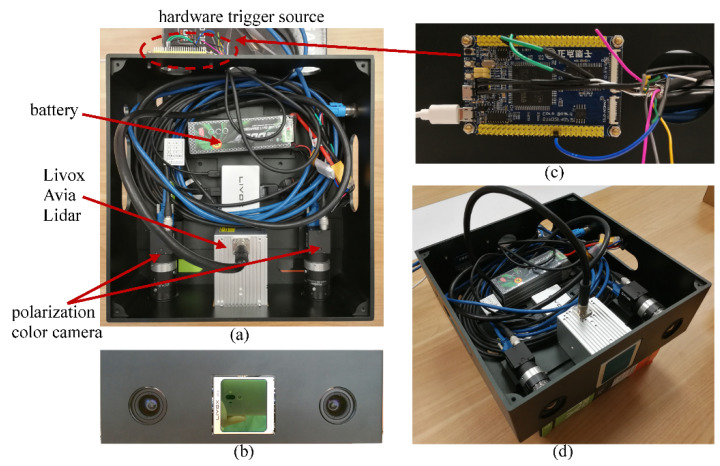
The hardware configuration: (**a**) experimental platform equipped with the polarization color stereo camera and the Livox Avia LiDAR. The LiDAR system is powered by a LiPo battery, commonly used in the quadcopter helicopter; (**b**) the front view of the hardware system; (**c**) the hardware trigger source, which is achieved using a STM32F407 development board. It realizes the synchronization of the cameras and the LiDAR based on the PPS synchronization methods; (**d**) the general view of the hardware system.

In our sensor fusion system, the Livox Avia LiDAR [12,13] and the FLIR polarization color cameras equipped with the Sony IMX250MYR CMOS [29] are utilized, as shown in Figure 2a. They are mounted on a frame fabricated by CNC machining, and their positions are fixed. The LiDAR system is powered by a LiPo battery, commonly used in the quadcopter helicopter. The hardware trigger source, which is achieved using a STM32F407 development board, is illustrated in Figure 2c. It realizes the synchronization of the stereo camera and the LiDAR based on the pulse per second (PPS) synchronization methods. The front view and general view of the hardware system are shown in Figure 2b,d, respectively.

The stereo camera subsystem is composed of two polarization color cameras (left and right) with global shutter, as illustrated in Figure 2a. Compared with the previous polarization information acquisition method, by rotating polarization filters [8] or adding different polarization direction filters before lens [9,10], the polarization camera, which has pixel-level wire grid polarizer structure on the CMOS, is employed in this paper. The polarization images with four different polarization directions, i.e., 0°, 45°, 90° and 135°, are gathered at the same time. Furthermore, an extra Bayer array is covered before the polarization mask, then the pixel-level aligned RGB and polarization information are all acquired. The polarization color camera used in this paper has the advantages of on-sensor polarimetry, high-speed global shutter readout, low-power package and compact size, which makes it ideal for a wide range of applications.

However, the camera detection results are very sensitive to time-varying environments and weather conditions. Moreover, the effective depth range of the stereo camera is limited, and the range accuracy is reduced when the depth exceeds the general detectable range. Conversely, the LiDAR based on the direct time of flight (dTOF) principle has high ranging accuracy and stable measurement results. Unlike the traditional mechanical LiDAR that uses high-grade optics and a rotating assembly to create a 360° field of view (FOV), the Livox Avia LiDAR based on prism scanning is employed in our paper [12,13]. With the advantage of low price and excellent performance, the Avia is an ideal selection. Nevertheless, the Livox Avia cannot precisely detect objects which are less than 1m away. The point cloud is distorted to a varying extent when the target object is within a range of 1 to 2 m. As a contrast, the range detection accuracy of the stereo camera is accurate in this scope. With the principle of multiple sensor data fusion, the performance of our system is excellent under different distances.

Before the data fusion, the sensors’ FOV also need to be considered. With the help of a 6mm focal length lens, the horizontal and the vertical FOV of the polarization color camera are 74.7° and 58.1°, respectively. Meanwhile, the horizontal and the vertical FOV of the Livox Avia LiDAR are 70.4° and 77.2°, respectively, under the mode of non-repetitive scanning pattern. The horizontal FOV of these two sensors is nearly equal, which can easily project the point cloud onto the color image to obtain the depth information of the corresponding data point. In terms of the software framework, our data fusion system is developed based on the Robotics Operating System (ROS) platform, and most nodes are coded in C++, except the node that touches on deep learning.

### 3.2. Object Detection and Recognition

In this subsection, we mainly introduce the object detection and recognition using the color images, the polarization images, the depth images and the point cloud.

#### 3.2.1. Object Recognition of the Color Images

**Figure 3:**
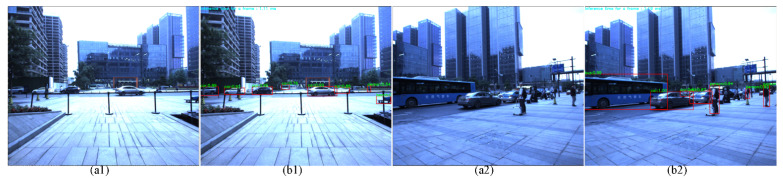
The object detection and recognition based on the color images: (**a1**,**a2**) the color images with zero-degree polarization; (**b1**,**b2**) the object detection and recognition using the YOLO V4 network, the detection and recognition results are labelled using the red bounding box. The class name and the confidence are put on the upper left corner of the bounding box.

The gray images with four different polarization directions are extracted from the raw image using the sampling method. Furthermore, the color images are obtained depending on the color convert. The objects are detected in different environments, as shown in Figure 3. The color images are illustrated in Figure 3(a1,a2), and the object detection and recognition results based on the YOLOv4 network [18] are presented in Figure 3(b1,b2). The detection and recognition results are labelled using the bounding box. The class name and the confidence are put on the upper left corner of the bounding box.

YOLOv4 is an efficient and powerful object detection model [18,30], which has excellent accuracy while maintaining a high processing frame rate. The YOLOv4 is composed of several parts, which mainly includes CSPDarknet53 backbone, SPP additional module, PANet path-aggregation neck and YOLOv3 (anchor based) head. The YOLOv4 model is trained on the Common Objects in Context (COCO) [31] dataset, and a number of additional designs and improvements are made in the training. The YOLOv4 network could achieve the real-time object detection with the graphics processing unit (GPU) performing, for instance, at about 1 ms per frame, running on a desktop computer (with I7 9700, 16G RAM, GeForce RTX 2080 Ti) for 1224 × 1024 input.

#### 3.2.2. Feature Extraction of the Polarization Images

In this section, the water surface detection method based on the polarization information is described. When the light reflects from the water surface, the polarization state is changed. Compared with the surrounding scenes, the polarization degree or polarization phases of the light reflected from water surface is obviously dissimilar.

Here, the degree of linear polarization (DoLP) [7] and the angle of linear polarization (AoLP) are introduced briefly, which are the significant elements to achieve the images segment. They are derived from Stokes Vectors S, which are composed of four parameters, i.e.,$S_{0}$, $S_{1}$, $S_{2}$, $S_{3}$. More precisely, $S_{0}$ represents the intensity of the light beam, $S_{1}$ stands for the difference between the intensities of the horizontally and vertically polarized pixels, and $S_{2}$ stands for the difference between the intensities of the 45° and 135° polarized pixels. $S_{3}$ is related to the circularly polarized light and is not involved in our work. They can be derived from:

(1) $$\{\begin{array}{l} S_{0}=I_{0}+I_{90}=I_{45}+I_{135},\\ S_{1}=I_{0}-I_{90},\\ S_{2}=I_{45}-I_{135}, \end{array}$$

where $I_{0}$, $I_{45}$, $I_{90}$ and $I_{135}$ represent the light intensity of the corresponding polarization direction. Here, DoLP and AoLP are described as:

(2) $$DoLP=\frac{\sqrt{S_{1}^{2}+S_{2}^{2}}}{S_{0}}$$

(3) $$AoLP=\frac{1}{2}\arctan\left(\frac{S_{2}}{S_{1}}\right)$$

According to Equations (2) and (3), the range of DoLP is from 0 to 1. For AoLP, it ranges from 0° to 180°. DoLP stands for the degree of linear polarization, and AoLP reflects objects’ silhouette information.

**Figure 4:**
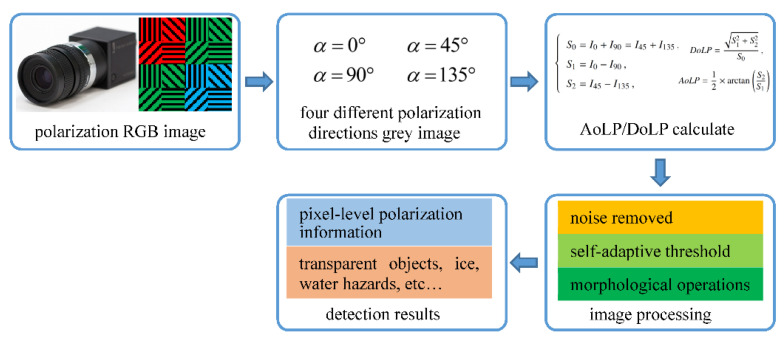
The processing procedure of polarization color images.

The water surface is segmented on the DoLP and AoLP images according to the difference in polarization degree or the polarization phases. The detailed processing procedure of the polarization color images is presented in Figure 4. When we obtain the raw data coming from the polarization color camera, four grey images with different polarization directions are extracted firstly. Then, the AoLP and DoLP images are calculated based on Equations (1)–(3). Moreover, the AoLP and DoLP images are segmented, with the basic procedures including noise removed, self-adaptive threshold and morphological operations. At last, the water hazards objects are detected successfully. At the same time, we also obtain the pixel-level polarization information.

#### 3.2.3. Object Detection of the Depth Images

**Figure 5:**
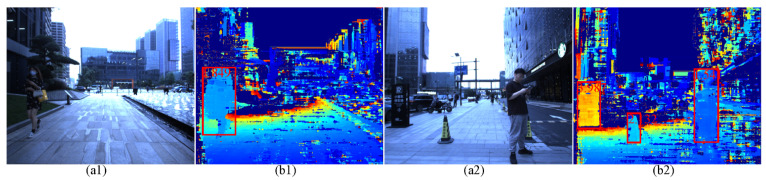
The object detection based on the depth images: (**a1**,**a2**) the color images with zero-degree polarization; (**b1**,**b2**) the depth images and the detection results. The detection results are labelled using the red bounding box.

The acquired left and right images are rectified based on the principle of the epipolar constraints. Then, the disparity images are obtained from the rectified images according to the semi-global matching (SGM) method. Furthermore, the depth images are achieved on the basis of the focal length and baseline parameters. The color images with zero-degree polarization, the depth images and the corresponding detection results are described in Figure 5(a1,a2) and (b1,b2), respectively. The detection results are indicated using the red bounding box. Herein, the MeanShift algorithm is applied to achieve the objects detection with the help of depth differences in the depth image.

The distance of the detected object is determined by the average depth in the red bounding box. The detection object coordinates in the pixel coordinate system are the center of the red bounding box. Then, the specific coordinates in the camera coordinate are obtained with the help of the camera intrinsic.

#### 3.2.4. Object Detection of the Point Cloud

**Figure 6:**
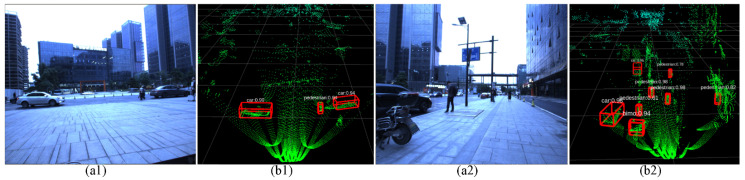
The object detection based on the point cloud: (**a1**,**a2**) the color images with zero-degree polarization; (**b1**,**b2**) the point cloud and the object detection and recognition results. The detection and recognition results are labelled using the red 3D bounding box. The class name and the confidence are put on the upper left corner of the bounding box.

When the non-repetitive scanning pattern of the Livox Avia LiDAR is employed, the scanning density is denser in the center of the FOV compared to the surrounding area. The point cloud shape looks like a flower. In the center of the FOV, within a radius of 10°, the point cloud density rivals traditional mechanical 32-line LiDAR sensor within 0.1 s. Over time, the point cloud density and coverage inside the FOV increase significantly and reveal more detailed information of the surroundings. Figure 6 reveals the objects detection results in different environments, while the color images are illustrated in Figure 6(a1,a2), and the LiDAR point cloud and the object detection results are presented in Figure 6(b1,b2). The detection results are labelled using the red 3D bounding box. The class name and the confidence are put on the upper left corner of the bounding box.

For the object detection in point clouds, the PointPillars [20] network is introduced. Additionally, the PointNets [32,33] are utilized in this network to learn the representation of point clouds organized in vertical columns. The features on pillars (vertical columns) of the point cloud are learned to predict 3D oriented boxes. It is an appropriate encoding for object detection in point clouds, which provides a trade-off between speed and accuracy. The network is trained using the Tensorflow, and the trained weights are fine-tuned. It can realize the recognition of the common objects, such as car, bus, truck and pedestrian. The running time of the PointPillars network is about 30ms per frame running on a desktop computer (with I7 9700, 16G RAM, GeForce RTX 2080 Ti), when the LiDAR integration time is set to 0.1s.

### 3.3. Calibration and Synchronization

In this multiple sensor data fusion system, it is necessary to achieve the coordinate calibration and the time synchronization.

**Figure 7:**
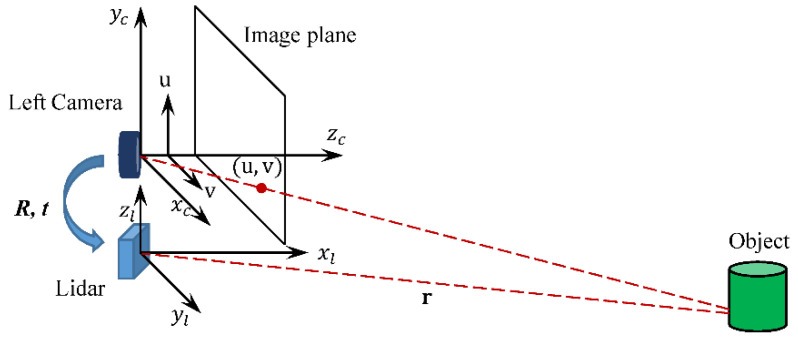
The left camera coordinate and LiDAR coordinate.

In this paper, the standard pinhole model is used for the polarization color camera. As shown in Figure 7, the $\left(x_{c},y_{c},z_{c}\right)$ is the camera coordinate, and (u,v) is the image plane coordinate. The relationship between them is described in Equation (4). The $f_{x}$, $f_{y}$, $c_{x}$ and $c_{y}$ are the x and y direction focal lengths and principle point coordinates, respectively, and the K is the matrix of intrinsic parameters.

(4) $$z_{c}\left[\begin{array}{c} u\\ v\\ 1 \end{array}\right]=K\left[\begin{array}{c} x_{c}\\ y_{c}\\ z_{c} \end{array}\right]=\left[\begin{array}{ccc} f_{x} & 0 & c_{x}\\ 0 & f_{y} & c_{y}\\ 0 & 0 & 1 \end{array}\right]\left[\begin{array}{c} x_{c}\\ y_{c}\\ z_{c} \end{array}\right]$$

For camera calibration, the chessboard images of the left and the right camera are acquired simultaneously. Then, the intrinsic and extrinsic parameters of these two cameras are obtained using calibration method of Zhang [34].

For the left camera and the LiDAR extrinsic parameters calibration, the Perspective-n-Point (PnP) method [35] and Ceres Solver library [36] are applied. When a target is detected by the LiDAR, its coordinate is $\left(x_{l},y_{l},z_{l}\right)$, as illustrated in Figure 7. The extrinsic matrix $M^{RT}$, which includes the rotation R and the translation T, between the left camera and the LiDAR, is obtained through

(5) $$z_{c}\left[\begin{array}{c} u\\ v\\ 1 \end{array}\right]=KM^{RT}\left[\begin{array}{c} x_{l}\\ y_{l}\\ z_{l}\\ 1 \end{array}\right]=K\left[\begin{array}{c} \begin{array}{cccc} m_{11} & m_{12} & m_{13} & m_{14} \end{array}\\ \begin{array}{cccc} m_{21} & m_{22} & m_{23} & m_{24} \end{array}\\ \begin{array}{cccc} m_{31} & m_{32} & m_{33} & m_{34} \end{array} \end{array}\right]\left[\begin{array}{c} x_{l}\\ y_{l}\\ z_{l}\\ 1 \end{array}\right]$$

where the extrinsic matrix $M^{RT}$ is made up of 12 elements.

**Figure 8:**
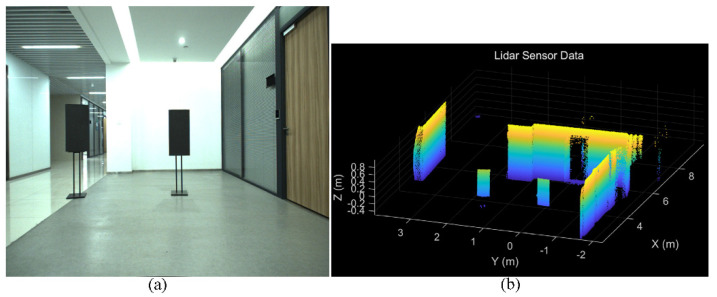
The processing procedure of the matched data point: (**a**) the color image; (**b**) the LiDAR point cloud data. In this case, two calibration boards made of low-reflectivity foam are used. In our project, we use the four corner points of the calibration board as the target points. The corresponding pixels on the image and LiDAR points are matched manually, then the matched data points are acquired.

In order to estimate the matrix $M^{RT}$, the corresponding data points are collected, and the linear least squares method is utilized with the help of the Ceres Solver library. In our project, we use the four corner points of the calibration board as the target points. In this case, two calibration board (about 0.5 × 1 m) made of low-reflectivity foam are used, as presented in Figure 8. The image and point cloud from different positions and different angles are extracted and matched manually. After calculation and analysis, the calibration matrix $M^{RT}$ is

(6) $$M^{RT}=\left[\begin{array}{c} \begin{array}{cccc} 0.0152864 & -0.999707 & -0.018759 & 0.074925 \end{array}\\ \begin{array}{cccc} -0.00409615 & 0.0186984 & \;-0.999817 & -0.000273555 \end{array}\\ \begin{array}{cccc} 0.999875 & 0.0153604 & -0.00380912 & 0.225082 \end{array} \end{array}\right]$$

**Figure 9:**
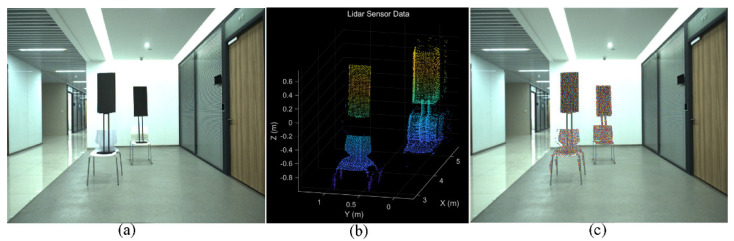
The object is detected by the camera and the LiDAR simultaneously: (**a**) the color image; (**b**) the LiDAR points cloud of the foam boards and chairs; (**c**) the LiDAR point cloud is projected on the color image.

After obtaining the extrinsic matrix, we project the point cloud on the corresponding color image to verify the calibration accuracy. The left camera image and the downsampling point cloud, which are out of the calibration data, are shown in Figure 9a,b, respectively. We select the point cloud of the foam boards and the chairs, then project them on the color image. The projection result is illustrated in Figure 9c. The projection result tells us the calibration accuracy is very high.

**Figure 10:**
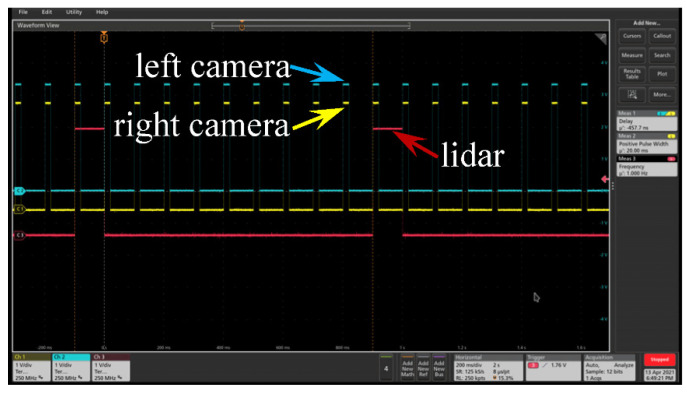
The stereo camera and LiDAR synchronization method. The trigger source signal received by the stereo camera and the LiDAR.

Except for the spatial coordinate calibration, the synchronization between cameras and LiDAR also needs to be considered. The camera is equipped with a 6-pin general-purpose input/output (GPIO) connector on the back of the case, which includes the hardware trigger interface. The camera is set to acquire images when the rising edge of the PPS arrives. Two independent but identical TTL waveforms with 10 Hz trigger the left and right cameras, respectively, as presented in Figure 10. Meanwhile, every time Livox LiDAR receives the rising edge of the PPS signal, it will set the point cloud time at the current moment to 0, and then restart timing until the next PPS pulse arrives. We use this feature to realize the synchronization of the PPS pulse to the LiDAR time. The LiDAR TTL trigger source with 1 Hz is also described in Figure 10. Simultaneously, the integration time of the Livox LiDAR is set to 0.1 s. Finally, we realize the synchronous acquisition of the multiple sensor with 10 Hz. Furthermore, from the oscilloscope, the time difference between these three hardware trigger waveforms is less than 10 microseconds, which guarantees time synchronization accuracy requirements for the ADAS.

### 3.4. Data Fusion

In our past work, we successfully established the track-to-track multiple sensor data fusion architecture [37]. Under this architecture, we actualized multiple sensor data fusion based on the joint integrated probabilistic data association (JPDA) algorithm and the Kalman filter [38]. By taking advantage of this architecture, we also previously performed the multiple sensor data fusion using the particle filter based on the principle of Monte Carlo sampling [16]. Furthermore, in this paper, we have realized the synchronization of the multiple sensor. The data fusion could be achieved frame by frame.

**Figure 11:**
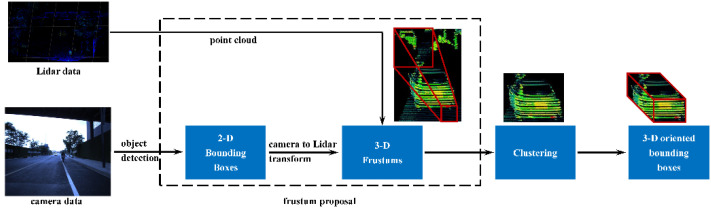
The processing procedure that estimating the depth information of the detected objects from 2D bounding boxes in image.

In most cases, the objects could be detected both in image and point cloud. LiDAR is skilled in obtaining the objects accurate depth information, while the camera produces rich and detailed information of the environment which is useful for object detection and classification. Accordingly, how to obtain the precise depth information of the objects in LiDAR point cloud using label data from the co-located camera with known LiDAR-to-camera calibration matrix is an important problem that needs to be solved in this multiple sensor data fusion system. The basic processing procedure that estimates the depth information of the detected objects from 2D bounding boxes in image is presented in Figure 11.

The 2D bounding boxes are generated on the color image by means of the YOLOv4 network, which is elaborated in Section 3.2.1. In order to generate corresponding cuboid bounding boxes in the point cloud from the 2D rectangular bounding boxes in the color image, the 3D regions are first proposed to reduce the search space for bounding box estimation. The corners of each 2D rectangular bounding box in the image are transformed into 3D lines with the help of the camera intrinsic matrix and the LiDAR-to-camera extrinsic matrix. Then, the frustum is formed by these 3D lines by the method of flaring out from the associated 2D bounding box in the opposite direction of the self-object. The LiDAR points in this area are divided into different clusters according to the Euclidean distance. The optimal cluster is estimated based on the size of these clusters, which is fitted with a 3D oriented bounding box. The cuboid boundaries are resolved by finding the minimum and maximum spatial extents in each direction. The distance of the detection object is decided by the center depth of the 3D bounding box. To improve the fitting accuracy of the 3D bounding boxes, the ground plane of the point plane is segmented and removed.

Nevertheless, the camera outlives its detection work when the overexposure or insufficient light happens. On the contrary, the LiDAR detection results keep stable without being affected by illuminative condition. We project the fitted 3D bounding boxes onto the image with the help of the LiDAR-to-camera extrinsic matrix. Then, the 2D bounding boxes, obstacle classifications, confidences and the corresponding coordinate information are labelled on the image.

## 4. Experiments

The experiments are designed and performed to verify the performance of our multiple sensor data fusion system. The hardware platform is connected to a portable PC (with I7-10875H, 16GB RAM, GeForce RTX 2060) by the USB port and network port. The portable PC is mainly responsible for the raw data acquired, including the polarization color images and the point cloud. These raw data are saved as the rosbag packages, which has a high performance and avoids the deserialization and reserialization of the messages. The data processing and fusion are executed by a desktop computer (with I7 9700, 16G RAM, GeForce RTX 2080 Ti), which includes object recognition of the color images, feature extraction of the polarization images, object detection of the depth images, object detection of the point cloud and data fusion. In this manner, the proposed multiple sensor data fusion system achieves the running speed of around 10 FPS.

### 4.1. Pixel-Level Aligned Polarization-Color-Depth Data Information

**Figure 12:**
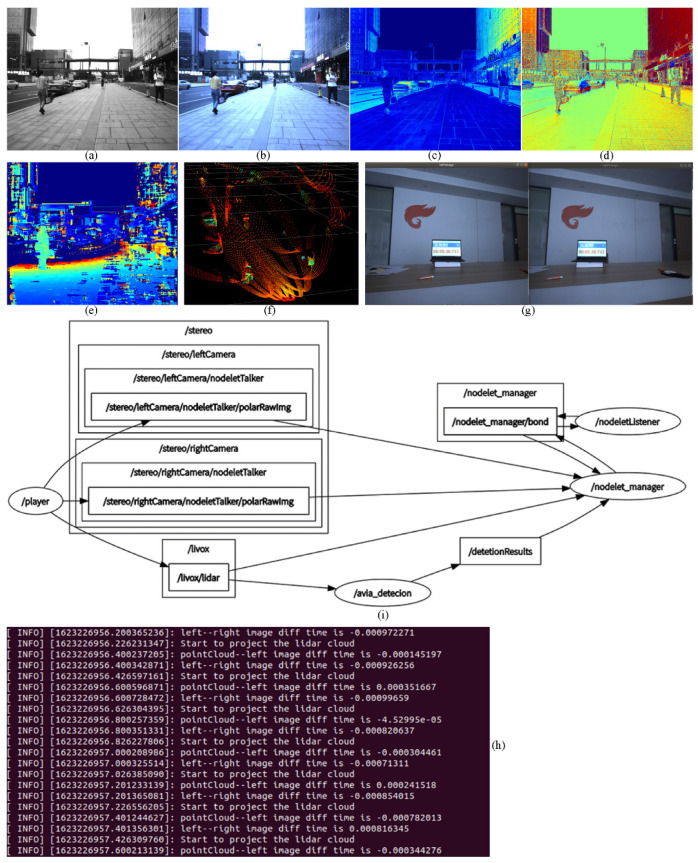
The data from multiple sensor and the synchronization accuracy of the hardware experimental platform: (**a**) the raw image of the left camera; (**b**) the color image with zero-degree polarization; (**c**) the normalization DoLP pseudo-color image (using opencv COLORMAP_JET); (**d**) the normalization AoLP pseudo-color image (using opencv COLORMAP_JET); (**e**) the stereo depth image, (**f**) the LiDAR point cloud; (**g**) the synchronization effect of the left and the right camera; (**h**) the synchronization effect of the left camera and the LiDAR, the unit is second; (**i**) the software framework of our data fusion system.

In order to verify the data obtained performance of our sensor data fusion system and the synchronization accuracy of the hardware experimental platform, the experiments are designed and performed as shown in Figure 12. The raw image of the left polarization color camera is presented in Figure 12a, which contains four grey sub-images with different polarization directions by the mosaic method. The grey image with zero-degree polarization is extracted, and converted into a color image, as described in Figure 12b. Similarly, we obtain the other three polarization grey images and the corresponding color images. According to Equations (2) and (3), the DoLP and AoLP images are calculated. For the sake of observation, the DoLP and AoLP images are normalized and processed with pseudo-color, as illuminated in Figure 12c,d, respectively. The polarization state of the slippery road surface and the glass curtain surface has significant difference compared with the surrounding environment.

Based on the principle of the epipolar constraints, the depth image could be created using the left and right rectified images, as shown in Figure 12e. Even though the effect range of the depth image is limited, it is sufficient for supplementing the distorted range of the LiDAR. The LiDAR point cloud synchronized with these images is shown in Figure 12f. In the center of the FOV within a radius of 10°, the point cloud density rivals traditional 32-line LiDAR sensor.

In terms of the synchronization accuracy, the stopwatch with millisecond precision on the web are shot by the left and right cameras simultaneously. The results are presented in Figure 12g. Thus, it can be seen that the time on the left and right images are the same, and the time synchronization accuracy of the left and right cameras is very high. As for the synchronization between the camera and the LiDAR, we evaluate it by calculating their timestamp difference. We subscribe the messages that come from the left camera node, right camera node and the LiDAR node at the same time. Then, the timestamp differences between these messages are obtained, as shown in Figure 12h. The time difference between left camera and the LiDAR system is very small, within 1 millisecond, which is caused by the time delay for different messages reaching the PC.

Finally, the software framework of our data fusion system is illuminated in Figure 12i, which is developed based on the ROS platform. We develop the ROS driver of the FLIR polarization color camera based on the nodelet to achieve automatic zero-copy transport and save time.

### 4.2. Slippery Road Surface and Puddles Detection

**Figure 13:**
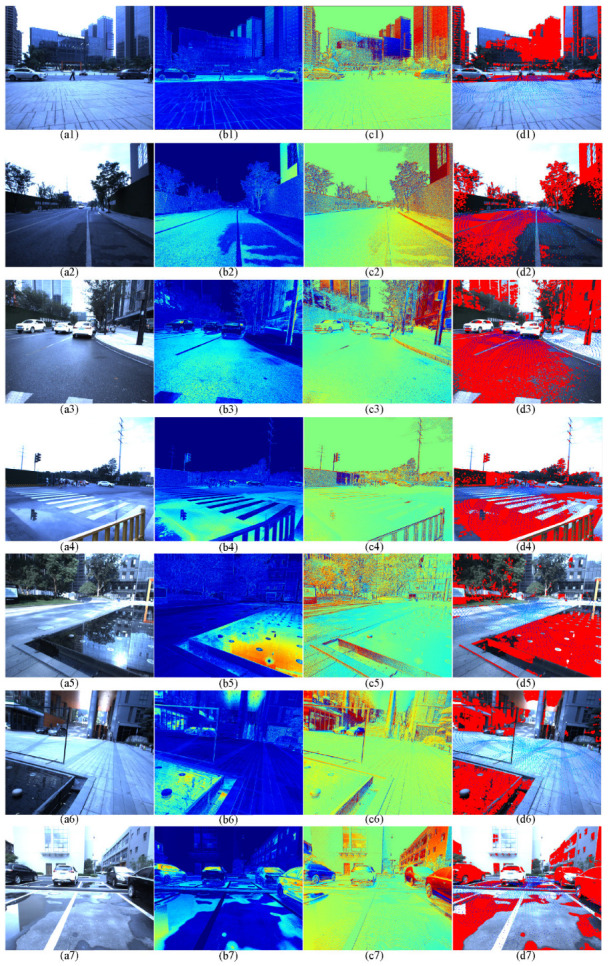
The slippery road surface and puddles detection results by the polarization information and the LiDAR point cloud: (**a1**–**a7**) the color image with zero-degree polarization; (**b1**–**b7**) the normalization DoLP pseudo-color images (using opencv COLORMAP_JET); (**c1**–**c7**) the normalization AoLP pseudo-color images (using opencv COLORMAP_JET); (**d1**–**d7**) the slippery road surface and puddles detection results, which are marked by the red mosaics and the projected LiDAR point cloud. Because of high reflectivity objects, such as glass curtain, widely applied in urban environment, the errors commonly appear in the detection results when the polarization information is used alone. Here, we segment the ground plane in the LiDAR point cloud, which uses the RANSAC plane fitting method with the constraint of ground surface normal vector, and project it on the corresponding image to assist the slippery road surface and puddles detection.

In this paper, we utilize the pixel-level polarization information and the LiDAR point cloud to achieve the detection of slippery road surfaces and puddles in the urban environment; the results are described in Figure 13. The color images with zero-degree polarization state are shown in Figure 13(a1–a7). The corresponding normalized DoLP and AoLP images are presented in Figure 13(b1–b7) and (c1–c7), respectively. They are processed with pseudo-color using the opencv COLORMAP_JET. Finally, the detection results are described in Figure 13(d1–d7). The slippery road surfaces and puddles are marked by the red mosaics and the projected LiDAR point cloud.

Several typical scenes containing slippery road surfaces are presented, and the corresponding color image with zero-degree polarization are shown in Figure 13(a1–a3). The normalized pseudo-color DoLP and AoLP images are illustrated in Figure 13(b1–b3) and (c1–c3), respectively. It can clearly be seen that the polarization state of the slippery road surface is obviously different from the surrounding environment. We could segment the color image with the help of this pixel-level polarization information to highlight the slippery parts on the road. The segmentation results are marked by the red mosaics, which are described in Figure 13(d1–d3). However, because of the high reflectivity of objects widely applied in urban environment, the errors commonly appear in the detection results when the polarization information is used alone. For instance, the glass curtain walls of the tall buildings in Figure 13(a1–a3) are also considered as the pavement and marked by the red mosaics. Here, we segment the ground plane in the LiDAR point cloud, which uses the RANSAC plane fitting method with the constraint of ground surface normal vector, and project it on the corresponding image to assist the slippery road surface detection. With the help of the pixel-level polarization information and the LiDAR point cloud, the slippery road surfaces are detected successfully.

Similarly, the different scenes with puddles are also experimented with. The color image with zero-degree polarization is presented in Figure 13(a5,a6). The puddles appear on the roadside, which is very dangerous for the cars driving. The normalized pseudo-color DoLP and AoLP images are shown in Figure 13(b5,b6,c5,c6), respectively. At last, the detection results are described in Figure 13(d5,d6). The puddles are jointly marked by the red mosaics and the projected point cloud.

We also collect some scenes that contain slippery road surfaces and puddles simultaneously, which are presented in Figure 13(a4,a7). There are puddles on the slippery road after the rain. The corresponding normalized pseudo-color DoLP and AoLP images are illustrated in Figure 13(b4,b7,c4,c7), respectively. Finally, the puddles and the slippery road surfaces are detected successfully at the same time, as Figure 13(d4,d7) described.

In order to analyze the slippery road surface and puddles detection results quantitatively, we collect the databases in the actual field, and give the statistical results, which are shown in Table 1. If the slippery road surface appears in the current scene, and it is detected successfully, it is counted as a true positive (TP); if it is classified as a non-aim object, it is counted as a false negative (FN). If the slippery road surface does not exist, and it is classified as a non-aim object, it is counted as a true negative(TN); if it is classified as a slippery surface, it is counted as a false positive (FP). Additionally, the same is true about the puddles detection results.

According to the statistical results from Table 1, the slippery road surface and the puddles detection results are basically stable, and the detection accuracy results are 98.91% and 98.71%, respectively. Furthermore, we also study the detection failure cases, and find that most error detection results appear when the incident light is affected by some buildings’ occlusion or reflection from certain angles causing the polarization information not to be calculated accurately.

It can be seen that the slippery road surface and puddles detection in the urban environment are achieved accurately and robustly utilizing the data fusion of the pixel-level polarization information and the LiDAR point cloud.

### 4.3. Objects Detection and Data Fusion

**Figure 14:**
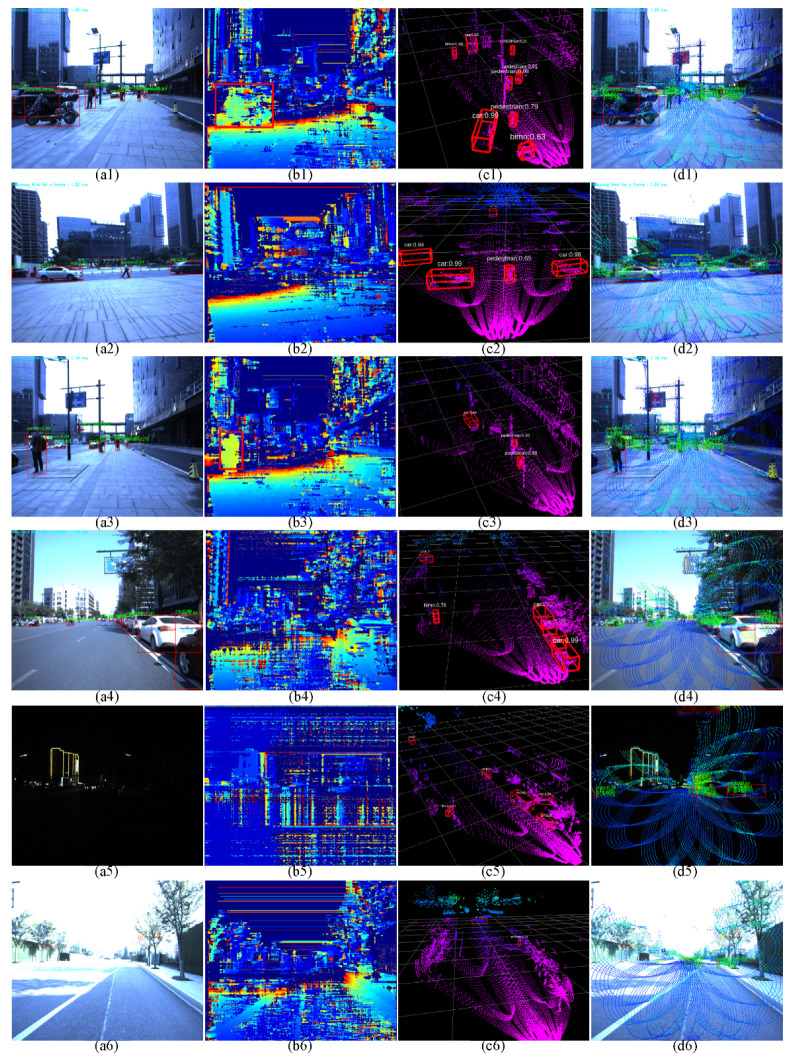
The objects detection and data fusion tests: (**a1**–**a6**) The color images with zero-degree polarization, and the object detection results using the YOLOv4 network. The detection and recognition results are labelled using the red bounding box. The class name and the confidence are put on the upper left corner of the bounding box. (**b1**–**b6**) The corresponding depth images, and the detection results using the MeanShift algorithm. The detection results are labelled using the red bounding box. (**c1**–**c6**) The LiDAR point cloud that is synchronized with the color image, and the detection results based on the PointPillars network. The results are labelled using the red 3D bounding box. The class name and the confidence are put on the upper left corner of the bounding box. (**d1**–**d6**) The data fusion results. The point cloud and the data fusion results are projected on the corresponding color image. The detected objects are labelled using the red bounding box. The class name, confidence and space coordinate are labelled on the upper left corner of the bounding box.

The object detection and data fusion tests are designed and performed with different surroundings, as shown in Figure 14. The color images with zero-degree polarization and the recognition results using the YOLOv4 are presented in Figure 14(a1–a6); the objects are detected with different environments. The depth images based on the principle of stereo camera and the corresponding detection results using the MeanShift algorithm are shown in Figure 14(b1–b6). The detection results are described by the red bounding boxes. The LiDAR point cloud and the corresponding detection results based on the PointPillars network are shown in Figure 14(c1–c6); the detection results are labelled using the red cuboid boxes. Finally, the data fusion results are illuminated in Figure 14(d1–d4). The class name, confidence and space coordinate are labelled on the upper left corner of the bounding box.

In scenario 1, a motorbike and several people and cars appear on the roadside in the afternoon when the sunlight is sufficient, which could be all detected and recognized in the color images using the YOLOv4 network, as Figure 14(a1) illustrated. Except for the motorbike, other objects vanish in the depth image, as they exceed the effective scope of the depth image. The depth image detection results are described in Figure 14(b1). By contrast, these objects are completely detected and recognized in the LiDAR point cloud based on the PointPillars network, as shown in Figure 14(c1). The detection results are labelled using the red 3D bounding box. The class name and the confidence are put on the upper left corner of the bounding box. Finally, the point cloud is projected on the color image, and the detection data are fused, as shown in Figure 14(d1). When the objects are detected by the depth image and the point cloud simultaneously, the fusion depth information is obtained using the Kalman filter. Nevertheless, some objects are out of the effective detection range of the depth image, thus the depth information of these objects are provided by the LiDAR point cloud. The bounding boxes, obstacle classifications, confidences and the corresponding coordinate information are labelled on the image. The detection results are enriched by the method of data fusion. At the same time, the effective detection range of the fusion system has been significantly expanded.

Owing to the limited perception range of the stereo camera, it needs to be able to detect dangerous obstacles from a distance. The LiDAR system is an appropriate sensor to achieve this function. In scenario 2, a person and four cars appear in the road, which are all detected and recognized in the color image, as shown in Figure 14(a2). However, these objects are out of the effective perception range of the depth images, and not detected, as presented in Figure 14(b2). Nevertheless, the LiDAR detection results are stable, which are described in Figure 14(c2). At last, the spatial coordinate information of these objects is decided by the point cloud based on the data fusion, which are illustrated in Figure 14(d2). In this sense, our fusion system extends the effective detection range compared to the detection with only stereo camera.

In some special complicated environments, the LiDAR loses sight of certain objects in some cases, due to the insufficient angular resolution or the sparse point cloud. In scenarios 3 and 4, cars and people have emerged on the roadside, and they are successfully detected and recognized in the color images, as shown in Figure 14(a3,a4). The nearest person in scenario 3 is found in the depth image, by contrast, most of them are not found, as described in Figure 14(b3,b4). What is more serious, the loss of targets has also been caused in the point cloud, as presented in Figure 14(c3,c4). On the contrary, the targets’ number and their categories could be confirmed in the high-resolution color images. While the last data fusion results are shown in Figure 14(d3,d4), the information about the objects are labelled on the images, which reveals the effectiveness of our approach. In this regard, with the help of data fusion, the detection results are enriched, the effective detection range is expanded, and the robustness of the prototype is improved.

In some special extreme environments, the LiDAR provides the last security guarantees. For instance, several cars come into our sight at nightfall, as shown in Figure 14(a5). These cars are not found in the color images owing to the low illumination. They are also not detected in the depth images because the quality of the depth image declines when the light is dim, when there is overexposure or when the perception distance exceeds the effective detection range. The detection results of the depth image are presented in Figure 14(b5). The LiDAR detection results, by contrast, are stable, as illustrated in Figure 14(c5). The last data fusion results are described in Figure 14(d5), which reveals the effectiveness of our approach even with low illumination. Similarly, the field test is also performed when the illumination is overexposed. The detection results of color image, depth image and point cloud are presented in Figure 14(a6), (b6) and (c6), respectively. The car and person are found due to the existence of the LiDAR. At last, the data fusion results are shown in Figure 14(d6). In this respect, our fusion system enhances the robustness of obstacle detection across different illumination conditions.

Experiments on object detection and data fusion show that multi-sensor data fusion significantly expands the effective detection range. Furthermore, the richer information and more robust performance are obtained compared with the single sensor. Compared with Gong et al. [21] and Zhang et al. [22] studies, which adopted similar technical solutions and employed the YOLOv3 to achieve the 2D objects detection in the data fusion framework, our proposed method improves the detection accuracy and calculation speed and achieves slippery road surface and puddles detection with the help of additional pixel-level polarization information.

## 5. Conclusions and Future Work

In this paper, we present a pixel-level aligned color-polarization-depth data fusion system, which comprises a stereo polarization color camera and a non-repetitive scanning LiDAR. The calibration and time synchronization between these sensors are established. The object detection and recognition of the color images based on the YOLOv4 network are achieved. The feature extraction of the depth image is realized using the MeanShift algorithm. The detection of slippery road surfaces and puddles is accomplished using the polarization information and the point cloud. The object detection in the point clouds is achieved using the PointPillars network. With the help of the multiple sensor data fusion, we obtain richer information on the detected targets. At the same time, compared with the detection using only stereo camera, the effective detection range of the fusion system has been expanded. Moreover, the detected results keep stable under diverse illumination conditions. This system could also be widely used in drones, robotics, surveillance and defense.

For future work, we plan to make more use of the pixel-level polarization information to form an attention fusion network that accomplishes multiple sensor data fusion at the low level. Additionally, we will also design a new network to improve the object detection precision and accuracy in the point cloud, especially when the point cloud is sparse.

## Figures and Tables

**Table 1:** The slippery road surface and puddles detection statistical results.

	Number of Samples	Detection Results		Accuracy(%)
slippery road surface	937 (positive)	TP:927	FN:10	98.91
	814 (negative)	FP:9	TN:805	
puddles	695 (positive)	TP:689	FN:6	98.71
	623 (negative)	FP:11	TN:612	

## Data Availability

Not applicable.
